# Exploring the prognostic impact of triglyceride-glucose index in critically ill patients with first-ever stroke: insights from traditional methods and machine learning-based mortality prediction

**DOI:** 10.1186/s12933-024-02538-y

**Published:** 2024-12-18

**Authors:** Yang Chen, Zhenkun Yang, Yang Liu, Yuanjie Li, Ziyi Zhong, Garry McDowell, Coleen Ditchfield, Taipu Guo, Mingjuan Yang, Rui Zhang, Bi Huang, Ying Gue, Gregory Y. H. Lip

**Affiliations:** 1 Liverpool Centre for Cardiovascular Science at University of Liverpool, Liverpool John Moores University and Liverpool Heart and Chest Hospital, William Henry Duncan Building, 6 West Derby Street, Liverpool, L7 8TX UK; 2 Department of Cardiovascular and Metabolic Medicine, Institute of Life Course and Medical Sciences, University of Liverpool, Liverpool, UK; 3 Department of Cardiology, Tianjin Medical University General Hospital, Heping District, Tianjin, People’s Republic of China; 4 Department of Cardiovascular Medicine, the Second Affiliated Hospital, Jiangxi Medical College, Nanchang University, Nanchang, Jiangxi People’s Republic of China; 5 Tianjin Research Institute of Anesthesiology and Department of Anesthesiology, Tianjin Medical University General Hospital, Tianjin, China; 6 Department of Musculoskeletal Ageing and Science, Institute of Life Course and Medical Sciences, University of Liverpool, Liverpool, UK; 7 School of Pharmacy and Biomolecular Sciences, Liverpool John Moores University, Liverpool, UK; 8 Department of Medicine for Older People, Whiston Hospital, Mersey and West Lancashire Teaching Hospitals NHS Trust, Prescot, UK; 9 Department of Cardiology, The First Affiliated Hospital of Chongqing Medical University, Chongqing, People’s Republic of China; 10 Department of Clinical Medicine, Danish Centre for Health Services Research, Aalborg University, 9220 Aalborg, Denmark

**Keywords:** Stroke, First-ever stroke, Insulin resistance, Triglyceride-glucose index, Intensive care unit, All-cause mortality

## Abstract

**Background:**

The incidence and mortality of first-ever strokes have risen sharply, especially in the intensive care unit (ICU). Emerging surrogate for insulin resistance, triglyceride-glucose index (TyG), has been linked to stroke prognosis. We aims to explore the relationships between TyG with ICU all-cause mortality and other prognosis, and to develop machine learning (ML) models in predicting ICU all-cause mortality in the first-ever strokes.

**Methods:**

We included first-ever stroke patients from the eICU Collaborative Research Database in 2014–2015 as the primary analysis cohort (then divided into training and internal validation cohorts) and from local hospital’s ICUs as the external validation cohort. Multivariate Cox proportional hazards models and restricted cubic spline analyses were used to evaluate the association between TyG and ICU/hospital all-cause mortality. Linear regression and correlation analyses were performed to examine the relationships between TyG with length of ICU/hospital stay and Glasgow Coma Score.

**Results:**

The primary analysis cohort included 3173 first-ever strokes (median age 68.0 [55.0–68.0] years; 63.0% male), while the external validation cohort included 201 first-ever strokes (median age 71.0 [63.0–77.0] years; 62.3% male). Multivariate Cox proportional hazards models revealed that the high TyG group (TyG ≥ 9.265) was associated with higher ICU (HR 1.92, 95% CI 1.38–2.66) and hospital (HR 1.69, 95% CI 1.32–2.16) all-cause mortality, compared with low TyG group (TyG < 9.265). TyG was also correlated with ICU length of stay (*r* = 0.077), hospital length of stay (*r* = 0.042), and Glasgow Coma Score (*r* = -0.132). TyG and other six features were used to construct ML models. The random forest model performed best in internal validation with AUC (0.900) and G-mean (0.443), and in external validation with AUC (0.776) and G-mean (0.399).

**Conclusion:**

TyG (optimal cut-off: 9.265) was identified as an independent risk factor for ICU and hospital all-cause mortality in first-ever strokes. The ML model incorporating TyG demonstrated strong predictive performance. This emphasises the importance of insulin resistance (with TyG as a surrogate measure) in the prognostic assessment and early risk stratification of first-time stroke patients.

**Supplementary Information:**

The online version contains supplementary material available at 10.1186/s12933-024-02538-y.

## Introduction

Stroke, the second leading cause of disability and mortality worldwide, has an average incidence of around 2,194 per 100,000 person-years and an age-standardized average mortality rate of approximately 818 per 100,000 person-years [1]. Further, about 13 million people, globally, have their first stroke annually, and the number of cases rises with increasing age [2]. The latest Global Burden of Disease study estimates that first-ever stroke increased by 70% globally from 1990 to 2019, and first-ever stroke-related deaths increased by 43% [3].

Patients experiencing their first stroke are a high-risk group whose management in the acute and recovery phases is further complicated by their relatively inexperienced awareness of the stroke and its prevention. Multiple studies have indicated that approximately 10–30% of patients require intensive care unit (ICU) treatment following their first-ever stroke [4]. The overall all-cause mortality of stroke patients admitted to ICU is about 15–20%, which is significantly higher than that in general wards [5]. Therefore, it is crucial to explore novel predictors that can help reduce mortality for patients with first-ever stroke in ICU, as well as to construct personalised prediction models to identify high-risk patients.

Insulin resistance (IR) refers to the reduced sensitivity of insulin-dependent organs and tissues to endogenous and exogenous insulin [6]. IR usually increases the risk of stroke by causing diabetes, dyslipidaemia, and hypertension, and is a significant risk factor for the development of cerebrovascular illness [7]. The current method to assess IR, including Homeostatic Model Assessment of IR and Hyperinsulinemic-Euglycemic Clamp, have limitations in the clinic, whereas the triglyceride-glucose index (TyG) has emerged as a reliable surrogate for IR because of its straightforward calculation and precision [8]. For cardiovascular diseases, TyG is a robust prognostic indicator for individuals with acute heart failure [9], and TyG was predictive of all-cause mortality in middle-aged and older hypertension patients during extended follow-up [10]. In a meta-analysis obtained from 18 studies, elevated TyG levels were related to increased risk of ischemic stroke (IS) in the general population, along with an increased likelihood of stroke recurrence and mortality [11]. One study suggested that elevated TyG levels were associated with ICU and hospital all-cause mortality in severely ill patients with IS (short-term survival did not vary significantly) [12], however, no study evaluates the effect of TyG on the prognosis (e.g. mortality and consciousness) of patients admitted to ICU with diagnosed first-ever stroke.

In recent years, machine learning (ML) is an emerging approach to constructing prediction model, and its application in healthcare has great potential and promise. TyG has been applied to the predictive models for the development of sepsis [13], coronary heart disease [14], nonalcoholic fatty liver disease [15], and diabetes mellitus (DM) [16]. However, studies assessing the prognostic role of the TyG in ICU patients with first-ever stroke are scarce, and no previous study has applied TyG measurement to clinical parameters using ML techniques to predict ICU all-cause mortality in the first-ever stroke patients.

Therefore, Our aims were two fold. (i) To investigate the association between TyG with ICU all-cause mortality and other prognosis outcomes of patients admitted to ICU with diagnosed first-ever stroke. (ii) To construct a ML model in predicting ICU all-cause mortality for such patients using TyG in combination with other clinical features.

## Methods

### Data sources

The primary analysis cohort in this study were consecutively and retrospectively obtained from the eICU Collaborative Research Database (EICU, version 2.0, recruited between 2014 and 2015) [17]. This database was a multicentre ICU database that collected clinical information on over 200,000 admissions to ICUs at 208 hospitals in the United States with a total of 335 ICU wards. EICU was constructed by the Laboratory for Computational Physiology, and included demographics, diagnostics, vital signs, laboratory measurements and treatments. Patient consent and ethical approval were not required as all data were anonymised. YC had access to the database and extracted all the data for this study after completing the Protecting Human Research Participants examination and the National Institutes of Health Web-based training course (Permission No.53753450). For the ML analysis, the primary analysis cohort was further split into *training* and *internal validation cohorts*.

### External validation cohort

To further externally validate the performance of the ML model, we consecutively and retrospectively recruited patients diagnosed with first-ever stroke admitted to the Neurology ICU and Comprehensive ICU of Tianjin Medical University General Hospital from October 2021 to June 2024. Baseline and clinical data of patients required to validate ML were extracted from the hospital’s electronic health record system.

### Ethics statement

Data collection in the EICU did not required the ethic approval, since this study was retrospective and without interventions for patients. And the safe harbour standards were assessed by an independent privacy expert (Privacert, Cambridge, MA) [Health Insurance Portability and Accountability Act Certification No. 1031219–2]. In this analysis, all data were anonymised and could therefore be exempted from local ethics review committee. For the external cohort, ethical approval was obtained from the Ethics Institutional Review Board of Tianjin Medical University General Hospital and informed consent was waived (No. IRB2024-YX-320-01).

### Inclusion and exclusion criteria

Inclusion criterion was all patients admitted to ICU and diagnosed with stroke. Exclusion criteria includes: (1) with history of stroke; (2) records of multiple ICU admissions or multiple hospitalizations; (3) without discharge records; (4) missing records of blood glucose and triglycerides on the first day; (5) age < 17years; (6) ICU length of stay < 6 h.

### Definitions of TyG, covariates and study outcomes

In our analysis, TyG was calculated as ln[glucose (mg/dL) × triglycerides (mg/dL)/2]. We extracted the following covariates: demographics variables (age, sex, race, and body mass index [BMI]), severity score at ICU admission (Acute Physiology and Chronic Health Evaluation IV [APACHE IV]), comorbidities (DM, hypertension, coronary artery disease, chronic kidney disease, heart failure, myocardial infarction, atrial fibrillation, respiratory failure [RFA], acute kidney injury [AKI], cirrhosis, sepsis), laboratory results at first day (total cholesterol, low-density lipoprotein cholesterol [LDL-C], and high-density lipoprotein cholesterol [HDL-C]), medications (antiplatelet agents, anticoagulants, and vasopressors), and interventions (thrombolysis, mechanical ventilation [MV]). Of these, BMI was calculated by weight(kg)/height(m)^2^, antiplatelet agents included clopidogrel, ticlopidine, aspirin, dipyridamole, and others; anticoagulants included heparin, coumadin, bivalirudin, argatroban, fondaparinux, and others; vasopressors included dopamine, epinephrine, norepinephrine, phenylephrine, and vasopressin. Repeated measurements of laboratory tests on the first day were collected only for the first value. Our primary study outcomes were ICU all-cause mortality, and secondary study outcomes were hospital all-cause mortality, length of ICU and hospital stay, and Glasgow Coma Score (GCS) and individual elements of GCS (eye opening, verbal response, motor response).

### Statistical analysis

Regarding missing values, according to Supplementary Table S1, we included the covariates with proportion of missing values less than 30%, and we used “miceforest” in Python to multiply interpolate the missing data.

For continuous variables, mean and standard deviation or median and interquartile range (IQR) were used as appropriate, and Student’s t-test or Mann–Whitney U test were applied to examine differences between groups. Categorical variables were evaluated the differences among groups using Fisher’s exact test or Chi [2] and expressed as counts and percentages.

Initially, we used the “survminer” package in R to find the optimal cut-off point of TyG. And we assessed the overall and nonlinear relationship between continuous TyG with ICU all-cause mortality using the restricted cubic spline analysis (RCS), and the reference point was set to the optimal cut-off point. Then, according to the categorised TyG based on the optimal cut-off point, Kaplan-Meier curves were plotted to assess survival differences. Then, we performed the multivariate Cox proprotion hazards models to evaluate the association between continuous or categorised TyG and ICU all-cause mortality (using the first group after the optimal cut-off division as the reference group), and the results were reported as hazard ratios (HR) and 95% confidence intervals (CI). In addition to considering the clinical significance of the covariates, we also examined correlations and multicollinearity between covariates to ensure the stability of the multivariate Cox proprotion hazards models. The results showed that total cholesterol was highly correlated with LDL-C (Pearson correlation coefficient [r] = 0.93). We further adjusted for different models. Model 1: unadjusted; Model 2: demographics (age, sex, race, and BMI), and severity score (APACHE IV); Model 3: further adjustments for comobidities (diabetes mellitus, hypertension, coronary artery disease, chronic kidney disease, heart failure, myocardial infarction, atrial fibrillation, RFA, AKI, cirrhosis, and sepsis), labotary tests (HDL-C and LDL-C), treatments (antiplatelet agents, anticoagulants, vasopressors, thrombolysis, and mechanical ventilation). Furthermore, subgroup analyses were performed to evaluate the persistence of the association between TyG and ICU all-cause mortality in different subgroups including age (≥ 60 and < 60years), sex, BMI (≥ 30 and < 30 kg/m^2^). Although DM status was important to TyG, the subgroup analysis of DM for ICU all-cause mortality was not performed due to limited numbers of ICU all-cause mortality events in DM patients. For the relationship between TyG and hospital all-cause mortlity, the same analytical process was performed as for ICU all-cause mortality, and the categorical value of TyG was consistent with the previous one. However, extra DM subgroup analysis was performed. Besides, Pearson correlation analysis and linear regression were performed to assess the relationship between TyG and other prognosis outcomes.

Additionally, we conducted three sensitivity analyses. First, considering the influence of systemic inflammation on the prognosis of cardiovascular disease, previous studies have shown that inflammatory biomarkers, such as C-reactive protein (CRP) and RDW, are associated with adverse outcomes of cardiovascular disease [18, 19]. However, in the EICU database, CRP values were missing in 97.1%, while the missing value proportion of RDW was less than 20.0% (Supplementary Table S1). Therefore, we extracted RDW data and added RDW to Model 3 to explore the relationship between TyG and mortality outcomes. Second, given that lipid-lowering treatments such as statins and fibrates significantly impact triglyceride levels, we extracted information on statins (atorvastatin, fluvastatin, lovastatin, pravastatin, simvastatin, imipenem-cilastatin, somatostatin) and fibrates (including clofibrate, fenofibrate). Then we conducted a sensitivity analysis by incorporating statin and fibrate use as confounders into Model 3 to assess whether lipid-lowering therapy modifies the relationships between TyG and mortality outcomes in patients with first-ever stroke populations. Third, as hypoglycaemic therapy (insulin and oral hypoglycaemic agents) directly affects glucose metabolism, it could potentially influence the TyG levels and subsequently affect outcomes. To account for this, we included hypoglycaemic therapy as an additional covariate in Model 3, allowing us to evaluate if hypoglycaemic therapy modified the associations between TyG and mortality outcomes.

Moreover, we performed univariate logistic regression and univariate linear regression analyses to assess the associations between each adjustment factor and the outcomes of first-ever stroke patients.

For the section on ML model in predicting ICU all-cause mortality for first-ever stroke patients, first, we divided the whole cohort into a *training cohort* (*N* = 2221) and an *internal validation cohort* (*N* = 952) by 7:3. Second, given the limited number of ICU deaths, feature pre-screening in the training set was performed using Boruta, which identifies features important to the prediction target by comparing the original features with randomly generated shadow features [20]. Third, we performed Pearson correlation tests and variance inflation factor tests on the selected features to avoid serious covariance or multicollinearity among the variables within the model. Fourth, the selected important features were entered into seven binary classification ML algorithms (including light gradient boosting machine, random forest [RF], logistic regression, support vector machine, multilayer perceptron, Gaussian Naive Bayes, and k-nearest neighbors) commonly used in the medical field for modelling in the training cohort, and using random search and manual fine-tuning with 5-fold cross-validation to obtain optimal hyperparameters for the each ML model (considering the imbalance of positive and negative events in the cohort, we applied class weights or synthetic minority oversampling technique in the ML models). Fifth, we verified the performance of all ML models in the internal validation cohort, plotting receiver operating characteristic (ROC) curves and decision curve analysis (DCA) curves, and comparing their performance against each other and APACHE IV based on several metrics including area under curve (AUC) with 95% CI, precision, recall, F1-score, G-mean, sensitivity, and specificity, to select the best ML model. Sixth, to make the ML model with complex internal structure more intuitive and understandable, we calculated the SHapley Additive exPlanation (SHAP) value for each feature in the best model, which was used to illustrate the different importance of the features on the outcome [21]. Moreover, dependent plot was plotted to observe the marginal effect of specific feature on the outcome of the ML model. Finally, to increase the utility of the ML model, we developed a web platform embedding it.

We performed all analysis by STATA (version 17, USA), SPSS (version 29, USA), R (version 4.2.3, Austria), Python (version 3.11.1, USA). A two-tailed *P-value* < 0.05 was used to evaluate statistical significance.

## Results

### Baseline characteristics and outcomes in cohort extracted from EICU

We included 3173 ICU patients with first-ever stroke from EICU (Supplementary Fig. S1). The median (IQR) age was 68.0 (55.0–68.0) years, 1664 (63.0%) were male, and 74.9% were White. The ICU and hospital all-cause mortality were 5.7% and 11.2%, respectively. Table 1 shows that ICU non-survivors had more males (62.4% vs. 51.8%, *P-value* = 0.006), RFA (49.2% vs. 13.5%, *P-value* < 0.001), AKI (9.4% vs. 4.1%, *P-value* = 0.002), and received significantly lower proportions of thrombolysis (8.8% vs. 24.4%, *P-value* < 0.001).

**Table 1:** Baseline characteristics of patients from the EICU

	ALL (*N* = 3173)	ICU survivors (*N* = 2992)	ICU non-survivors (*N* = 181)	*P*-value
Age, years	68.00 (55.00, 68.00)	68 (57.00, 78.00)	67.00 (54.00, 77.00)	0.206
Male, n (%)	1664 (63%)	1551 (51.8%)	113 (62.4%)	0.006
BMI (kg/m^2^)	27.72 (24.21, 32.67)	27.73 (24.21, 32.54)	27.46 (24.18, 31.50)	0.399
Race, n (%)				0.154
African American	368 (11.6%)	339 (11.3%)	29 (16.0%)	
Caucasian	2377 (74.9%)	2247 (75.1%)	130 (71.8%)	
Other/Unknow	428 (13.5%)	406 (13.6%)	22 (12.2%)	
APACHE IV	47.00 (34.00, 62.00)	46.00 (34.00, 59.00)	80.00 (62.00, 98.00)	< 0.001
Comorbidities, n (%)				
Diabetes mellitus	312 (9.8%)	291 (9.7%)	21 (11.6%)	0.439
Hypertension	1002 (41.8%)	941 (31.5%)	61 (33.7%)	0.564
Coronary artery disease	90 (2.8%)	82 (2.7%)	8 (4.4%)	0.170
Chronic kidney disease	102 (3.2%)	96 (3.2%)	6 (3.3%)	0.829
Heart failure	88 (2.8%)	81 (2.7%)	7 (3.9%)	0.347
Myocardial infarction	64 (2.0%)	57 (1.9%)	7 (3.9%)	0.092
Atrial fibrillation	314 (9.9)	299 (10.0)	15 (8.3)	0.523
Respiratory failure	494 (15.6)	405 (13.5%)	89 (49.2%)	< 0.001
Acute kidney injury	139 (4.4%)	122 (4.1%)	17 (9.4%)	0.002
Cirrhosis	10 (0.3)	6 (0.2)	4 (2.2)	0.002
Sepsis	82 (2.6%)	71 (2.4%)	11 (6.1%)	0.006
Laboratory results at first day				
LDL-C, mg/dL	88.00 (67.00, 115.00)	89.00 (68.00, 115.00)	78.00 (52.00, 107.50)	< 0.001
HDL-C, mg/dL	93.00 (34.00, 54.50)	43.00 (35.00, 55.00)	39.00 (30.00, 53.50)	0.001
TC, mg/dL	158.00 (131.00, 189.00)	159.00 (132.00, 190.00)	152.00 (116.00, 183.00)	0.004
Triglycerides, mg/dL	103.00 (73.00, 150.00)	73.00 (56.00, 103.00)	80.50.00 (56.20, 121.00)	0.004
Glucose, mg/dL	121.00 (101.00, 153.00)	101.00 (89.00, 119.00)	120.00 (103.20, 151.00)	< 0.001
Medications, n (%)				
Antiplatelet agents	337 (10.6)	323 (10.8)	14 (7.7)	0.215
Anticoagulants	197 (6.2)	188 (6.3)	9 (5.0)	0.633
Vasopressors	147 (4.6%)	119 (4.0%)	28 (15.5%)	< 0.001
Interventions, n (%)				
Thrombolysis	747 (23.5%)	731 (24.4%)	16 (8.8%)	< 0.001
Mechanical ventilation	582 (18.3)	454 (15.2%)	128 (70.7%)	< 0.001
TyG	8.77 (8.37, 9.22)	8.74 (8.36, 9.19)	9.12 (8.64, 9.66)	< 0.001

Abbreviations: APACHE IV, Acute Physiology and Chronic Health Evaluation IV; BMI, body mass index; EICU, eICU Collaborative Research Database; HDL-C, high-density lipoprotein cholesterol; ICU, intensive care unit; LDL-C, low-density lipoprotein cholesterol; TC, total cholesterol

Besides, the optimal cut-off point for ICU all-cause mortality was determined as 9.265 (Supplementary Fig. S2), so TyG was categorised as high TyG (TyG < 9.265) and low TyG (TyG ≥ 9.265). The high TyG group had higher BMI, higher LDL-C, and lower HDL-C (shown in Supplementary Table S2). And in Supplementary Table S3, high TyG group had higher ICU all-cause mortality compared to low TyG group (11.1% vs. 4.1%, *P-value* < 0.001), hospital all-cause mortality followed the same pattern (17.8% vs. 9.2%, *P-value* < 0.001). Additionally, high TyG group had significant longer ICU length of stay (*P-value* < 0.001), but the hospital length of stay did not differ statistically. For the GCS and its compositions, there were also statistical differences (*P-value* < 0.001).

### Association of TyG and ICU all-cause mortality in first-ever stroke patients

**Fig. 1:**
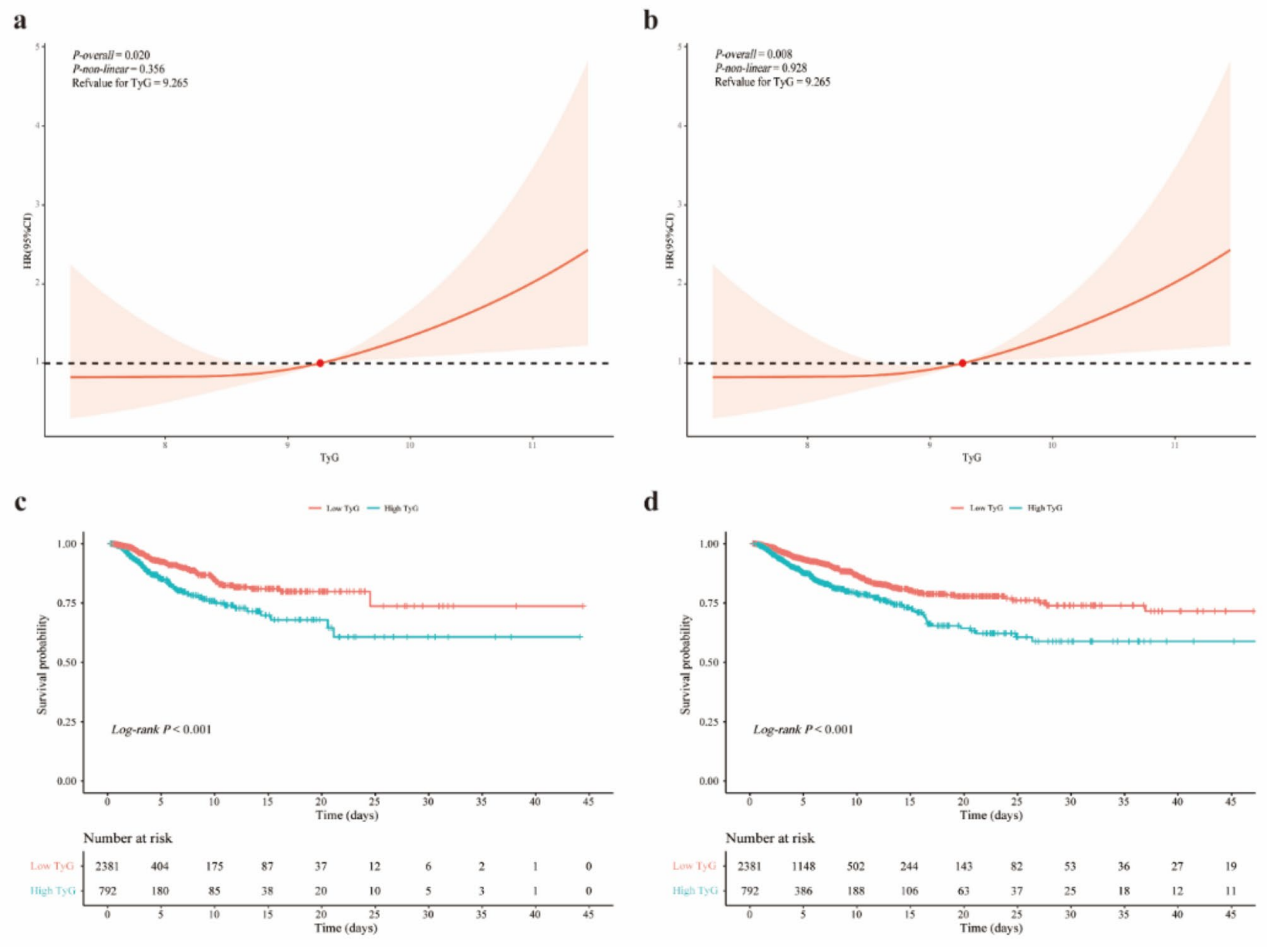
Restricted cubic spline analysis and Kaplan-Meier curves for ICU all-cause mortality (**a** & **c**) and hospital all-cause mortality (**b** & **d**). Low TyG: TyG < 9.265; high TyG: TyG ≥ 9.265. ICU, intensive care unit; HR, hazards ratio; TyG, triglyceride-glucose index

The results of the RCS analysis demonstrated a correlation between TyG and ICU all-cause mortality (*P-overall* = 0.020), but not a non-linear relationship (*P-non-linear* = 0.356) [Fig. 1a]. The Kaplan-Meier curve showed significant difference in survival probability between the high TyG and low TyG groups (*log-rank P* < 0.001) [Fig. 1c]. Based on Table 2, after adjusting all potential confounders, the results of multivariate Cox proprotional hazards models revealed that the continuous TyG was associated with ICU all-cause mortality in the first-ever stroke patients (HR: 1.34, 95% CI: 1.07–1.67). And high TyG group had higher risk of ICU all-cause mortality (HR: 1.92, 95% CI: 1.38–2.66), compared to low TyG group. Furthermore, subgroup analysis revealed no significant interaction between TyG and the subgroups on ICU all-cause mortality (Supplementary Figure S3a).

**Table 2:** Association between TyG and mortality in the first-ever stroke patients from the EICU

			Model I		Model II		Model III	
	*N*	Events, *n* (%)	HR (95% CI)	*P*-value	HR (95% CI)	*P*-value	HR (95% CI)	*P*-value
**ICU mortality**								
Continuous TyG			1.48 (1.23, 1.77)	< 0.001	1.41 (1.16, 1.73)	< 0.001	1.36 (1.08, 1.71)	0.010
Categorised TyG								
TyG < 9.265	2436	99 (4.1)	*Reference*		*Reference*		*Reference*	
TyG ≥ 9.265	737	82 (11.1)	2.20 (1.64, 2.96)	< 0.001	1.96 (1.45, 2.66)	< 0.001	1.92 (1.38, 2.67)	< 0.001
**Hospital mortality**								
Continuous TyG			1.37 (1.19, 1.56)	< 0.001	1.39 (1.20, 1.61)	< 0.001	1.31 (1.10, 1.55)	0.002
Categorised TyG								
TyG < 9.265	2436	223 (9.2)	*Reference*		*Reference*		*Reference*	
TyG ≥ 9.265	737	131 (17.8)	1.82 (1.46, 2.26)	< 0.001	1.82 (1.46, 2.28)	< 0.001	1.69 (1.32, 2.15)	< 0.001

Model I: Unadjusted

Model II: Adjusted by demographics (age, sex, race, and BMI), and severity score (APACHE IV)

Model III: Model II further adjusted by comobidities (diabetes mellitus, hypertension, coronary artery disease, chronic kidney disease, heart failure, myocardial infarction, atrial fibrillation, respiratory failure, acute kidney injury, cirrhosis, and sepsis), labotary tests (HDL-C and LDL-C), treatments (antiplatelet agents, anticoagulants, vasopressors, thrombolysis, and mechanical ventilation)

Abbreviations: APACHE IV, Acute Physiology and Chronic Health Evaluation IV; BMI, body mass index; EICU, eICU Collaborative Research Database; HDL-C, high-density lipoprotein cholesterol; ICU, intensive care unit; LDL-C, low-density lipoprotein cholesterol

### Association of TyG and hospital all-cause mortality in first-ever stroke patients

The results of the RCS analysis demonstrated a correlation between TyG and ICU all-cause mortality (*P-overall* = 0.008), but not a non-linear relationship (*P-non-linear* = 0.928) [Fig. 1b]. The Kaplan-Meier curve showed significant difference in survival probability between the high TyG and low TyG groups (*log-rank P* < 0.001) [Fig. 1d]. According to the findings in Table 2, the multivariate Cox proprotional hazards models, after adjusting all potential confounders, demonstrated that the continuous TyG was associated with hospital all-cause mortality in the first-ever stroke patients admitted to ICU (HR: 1.30, 95% CI: 1.10–1.54). And compared to low TyG group, high TyG group had higher risk of hospital all-cause mortality (HR: 1.69, 95% CI: 1.32–2.16). Furthermore, subgroup analysis revealed no significant interaction between TyG and the subgroups on hospital all-cause mortality (Supplementary Fig. S3b).

### Association of TyG with other outcomes in first-ever stroke patients

For the duration of ICU or hospitalization stay (Supplementary Fig. S4 & Supplementary Table S4), TyG showed a slight but statistically significant positive correlation with duration of ICU stay (*r* = 0.077, *P* < 0.001) and hospitalization stay (*r* = 0.042, *P* = 0.021), suggesting a positive association between higher TyG levels and prolonged durations of both ICU and hospitalization stay.

The linear regression analysis indicated that for each unit increase in TyG, there was a corresponding increase of 0.092 days (*P* < 0.001) in ICU stay and 0.054 days (*P* = 0.002) in hospital stay. For the conscious function outcomes (Supplementary Fig. S4 & Supplementary Table S4), TyG was negatively associated with the GCS score (*r* = − 0.132, *P* < 0.001), number of eye openings (*r* = − 0.128, *P* < 0.001), verbal responses (*r* = − 0.107, *P* < 0.001) and motor responses (*r* = − 0.116, *P* < 0.001). These negative correlations indicate that the higher the TyG value, the worse the neurological condition indicated by the GCS subscores. The linear regression analyses also revealed that with each unit increase in TyG, there was a corresponding decrease of 0.131 points in the GCS score (*P* < 0.001), 0.128 points in the number of eye-openings (*P* < 0.001), 0.113 points in the verbal response (*P* < 0.001), and 0.106 points in the motor response (*P* < 0.001). Overall, TyG was significantly associated with both hospital stay length and neurological assessment outcomes in patients, with higher TyG predicting longer hospital stays and poorer neurological function.

### Sensitivity analyses for relationships of TyG and mortality outcomes

After further adjustment for RDW in Model 3 (Supplementary Table S5), each unit increase in continuous TyG was associated with an elevated risk of ICU (HR: 1.34, 95% CI: 1.07–1.68) and hospital all-cause mortality (HR: 1.33, 95% CI: 1.12–1.57). Compared with TyG < 9.265, TyG ≥ 9.265 was associated with a higher risk of ICU (HR: 1.94, 95% CI: 1.39–2.69) and hospital all-cause mortality (HR: 1.75, 95% CI: 1.36–2.23).

When adjusting for lipid lowering therapy (statins and fibrates) in Model 3 (Supplementary Table S6), each unit increase in continuous TyG was related to an elevated risk of ICU (HR: 1.34, 95% CI: 1.07–1.67) and hospital all-cause mortality (HR: 1.30, 95% CI: 1.10–1.54). Similarly, TyG ≥ 9.265 was linked to higher risks of ICU mortality (HR: 1.93, 95% CI: 1.39–2.69) and hospital all-cause mortality (HR: 1.70, 95% CI: 1.33–2.17) compared to TyG < 9.265.

After accounting for hypoglycaemic therapy (insulin and oral hypoglycaemic agents) in Model 3 (Supplementary Table S7), each unit increase in continuous TyG was correlated with elevated risks of ICU (HR: 1.33, 95% CI: 1.06–1.66) and hospital all-cause mortality (HR: 1.29, 95% CI: 1.09–1.53). TyG ≥ 9.265 remained significantly associated with increased risks of ICU (HR: 1.90, 95% CI: 1.37–2.64) and hospital all-cause mortality (HR: 1.68, 95% CI: 1.31–2.14) compared to lower TyG levels.

### Exploratory analysis of the associations between adjustment factors and outcomes

Supplementary Table S8 shows the results of the associations between each adjustment factor and the outcomes of patients with first-ever stroke. MV (HR: 4.48, 95% CI: 3.22–6.23), APACHE IV (HR: 1.03, 95% CI: 1.03–1.04), RFA (HR: 1.91, 95% CI: 1.40–2.59) and cirrhosis (HR: 3.06, 95% CI: 1.13–8.26) were significantly associated with ICU all-cause mortality. In the analysis of hospital all-cause mortality, major associated factors were similar, including MV (HR: 4.23, 95% CI: 3.41–5.24), APACHE IV (HR: 1.03, 95% CI: 1.03–1.04), RFA (HR: 2.54, 95% CI: 2.04–3.16), cirrhosis (HR: 5.57, 95% CI: 2.76–11.20), thrombolysis (HR: 0.65, 95% CI: 0.47–0.89) and vasopressors (HR: 1.88, 95% CI: 1.37–2.57). Moreover, MV (β = -5.22, *P-value* < 0.001), vasopressors (β = -2.26, *P-value* < 0.001), insulin (β = -0.48, *P-value* = 0.004), RFA (β = -4.38, *P-value* < 0.001), sepsis (β = -2.63, *P-value* < 0.001) and AKI (β = -1.78, *P-value* < 0.001) were associated with poorer GCS, while thrombolysis (β = 1.18, *P-value* < 0.001) was associated with better GCS.

### Feature selection and collinearity tests

Supplementary Fig. S5 demonstrates that seven features have been selected by Boruta to be important for ICU all-cause mortality, with TyG considered as the third important feature, and other selected features are MV, RFA, HDL-C, vasopressor, sex, and LDL-C. Pearson correlation and variance inflation factor tests demonstrated no strong correlation or multicollinearity between them (Supplementary Fig. S6).

### Model construction and evaluation

Using these seven selected features, we built predictive models for ICU all-cause mortality in critical ill patients with first-ever stroke in the training cohort. The best hyperparameters of the MLs following five-fold cross-validation are shown in Supplementary Table S9.

**Fig. 2:**
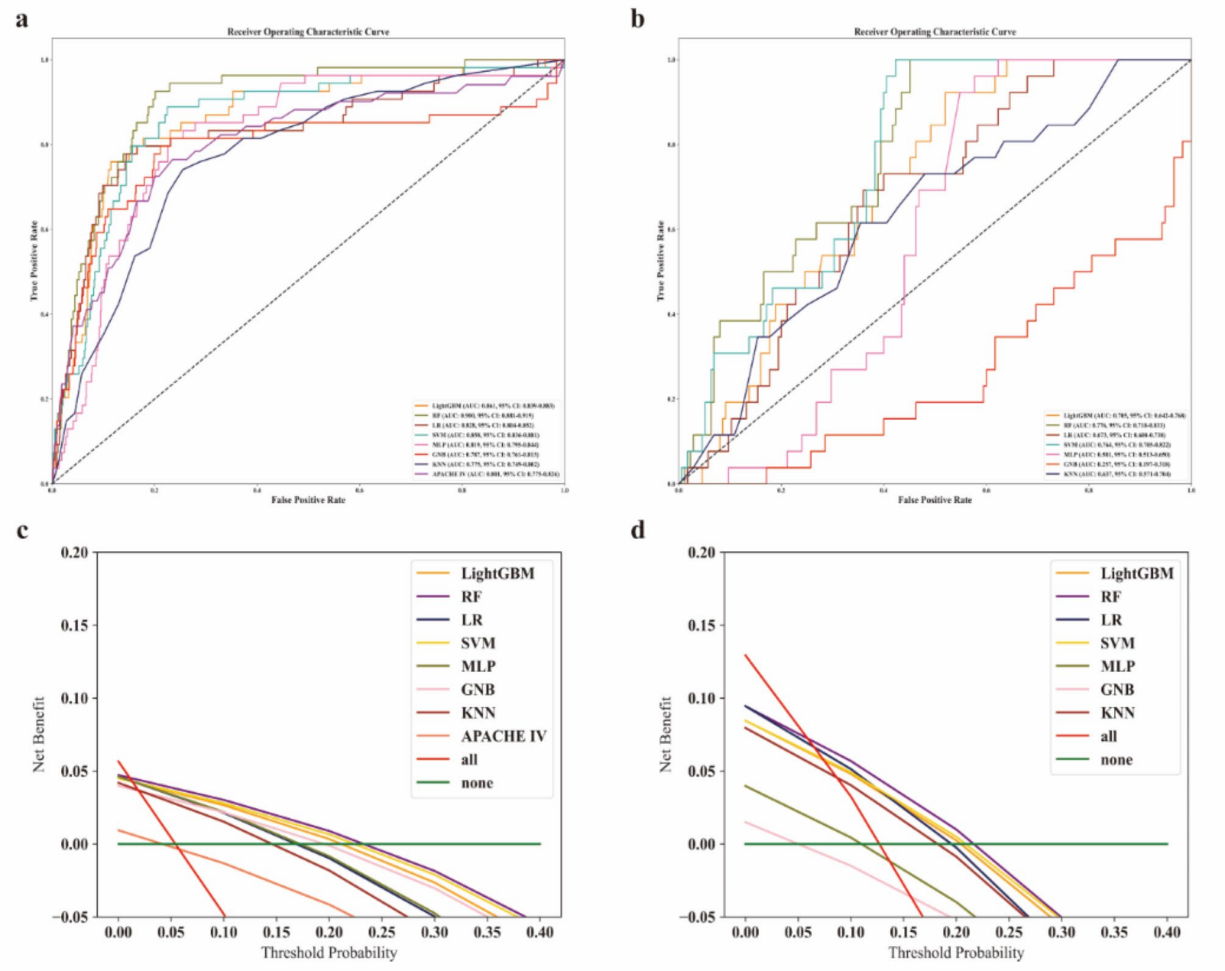
Evaluation the performance of all machine learning models with ROC and DCA in the *internal validation cohort* (**a** & **c**) and *external validation cohort* (**b** & **d**). Red “all” indicates that all patients received treatment, green “none” indicates that all patients did not receive treatment, and the area under each model is the net clinical benefit from applying the predictive model. APACHE IV, Acute Physiology and Chronic Health Evaluation IV; AUC, area under curve; CI, confidence interval; DCA, decision curve analysis; GNB, Gaussian Naive Bayes; KNN, k-nearest neighbors; LightGBM, light gradient boosting machine; LR, logistic regression; MLP, multilayer perceptron; RF, random forest; ROC, ceceiver operating characteristic curve; SVM, Support Vector Machine

**Fig. 3:**
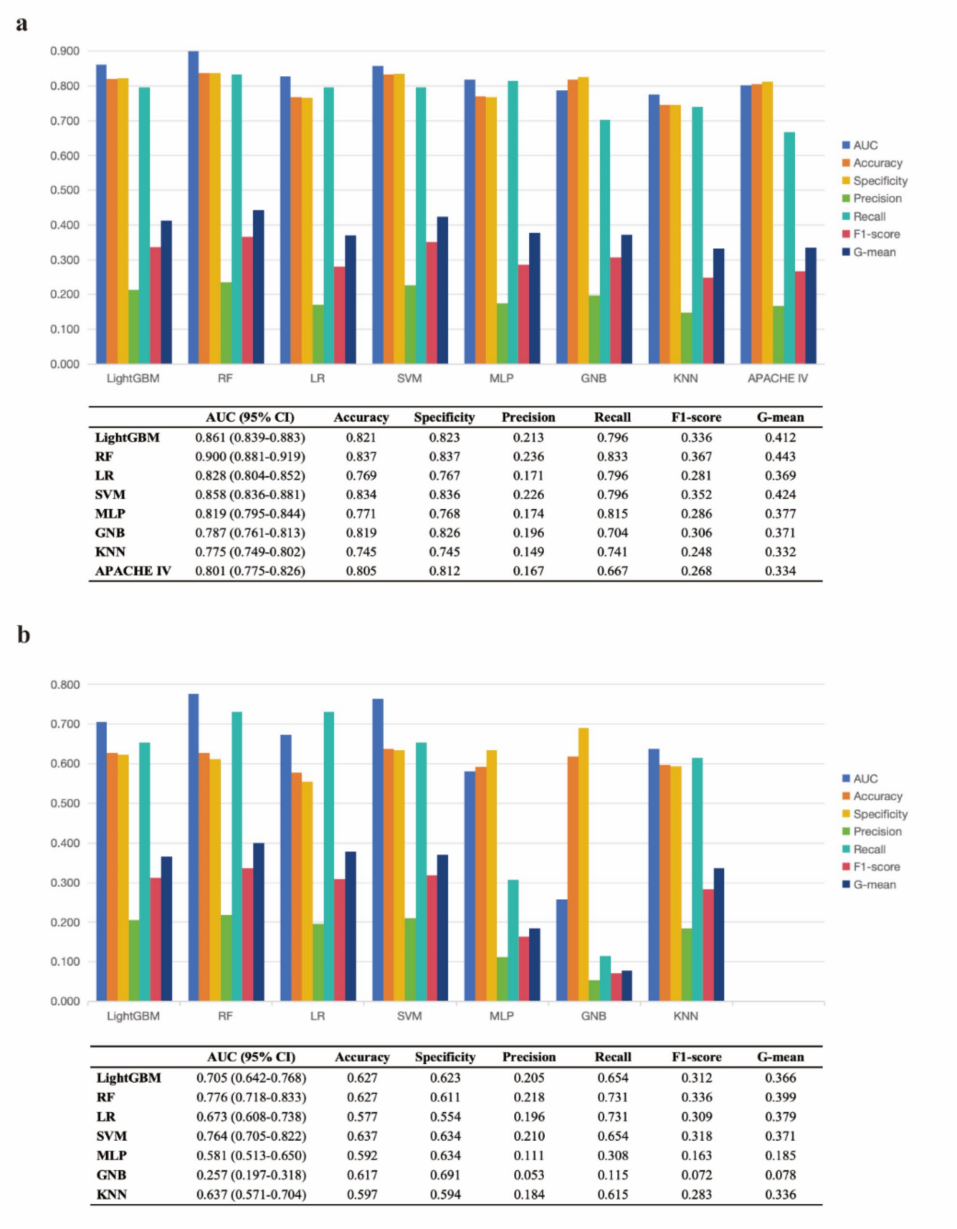
Evaluation the performance of all machine learning models with other metrics, and dependence plot of the effect of TyG for RF in the *internal validation cohort* (**a** & **c**) and *external validation cohort* (**b** & **d**). APACHE IV, Acute Physiology and Chronic Health Evaluation IV; AUC, area under curve; GNB, Gaussian Naive Bayes; KNN, k-nearest neighbors; LightGBM, light gradient boosting machine; LR, logistic regression; MLP, multilayer perceptron; RF, random forest; SVM, Support Vector Machine

According to the plots of ROC and DCA for the ML models in the *internal validation cohort* (Fig. 2a and c), only light gradient boosting machine, RF and logistic regression had higher AUCs than APACHE IV, with RF obtaining the highest AUC (0.900, 95% CI 0.881–0.919) and RF having the greatest net clinical benefit. Further calculating the other metrics (Fig. 3a), RF also had the highest G-mean (0.443) and F1 score (0.367).

In the *external validation cohort*, we recruited 201 first-ever stroke patients admitted to ICU, the baseline characteristics were shown in Supplementary Table S10. The RCS analysis of TyG and ICU all-cause mortality was presented in Supplementary Fig. S7, which was similar to the findings in the cohort extracted from EICU (*P-overall* = 0.010, *P-non-linear* = 0.376). However, there was no correlation analysis between TyG and GCS because of the limited recording of GCS (< 25%) in the *external validation cohort*. For the plots of ROC and DCA for the ML models in the *external validation cohort* (Fig. 2b and d, and Fig. 3b), RF still had the highest AUC (0.776, 95% CI 0.718–0.833), G-mean (0.399), F1 score (0.336), and had the greatest net clinical benefit.

### Feature importance and web platform

**Fig. 4:**
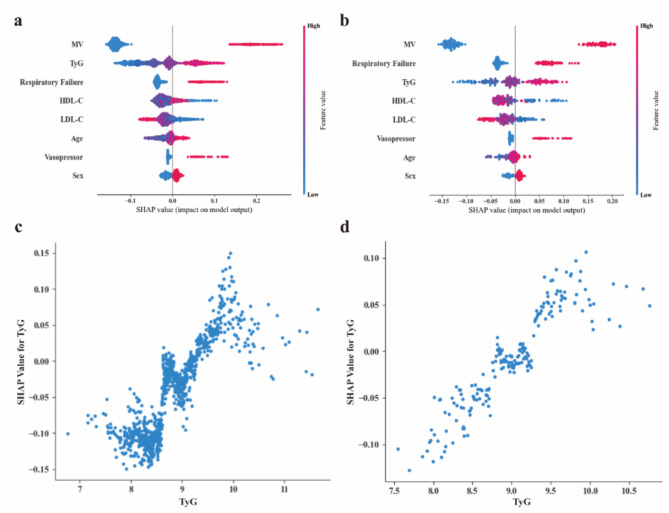
SHAP value and importance for each feature of our random forest model in the *internal validation cohort* (**A**) and *external validation cohort* (**B**). HDL-C, high-density lipoprotein cholesterol; LDL-C, low-density lipoprotein cholesterol; MV, mechanical ventilation; SHAP, SHapley Additive exPlanations; TyG, triglyceride-glucose index

Figure 4a and b show that the order from top to bottom on the Y-axis indicates the importance of all features in the RF for the ICU all-cause mortality, where the top three are: MV, TyG and RFA (TyG is second and third in the *internal validation cohort* and *external validation cohort*, respectively). Figure 4c and d shows that as the TyG becomes higher, the greater the positive contribution of TyG to the predicted value. Then, we embedded RF in a web platform with an easy-to-use interface containing inputs corresponding to the seven features within the model (http://162.62.58.247:3030/). By entering information specific to a particular patient, the probability of its outcome occurring would be output. For example, in Supplementary Fig. S8, a 65-year male patient received MV and vasopressor, his TyG, LDL-C and HDL-C on the first day of ICU were 9.5, 90 mg/dL, and 55 mg/dL, respectively, then he has a predicted probability of 77% and is therefore at high risk of ICU all-cause mortality.

## Discussion

Our multicenter study explored the association between TyG and prognostic outcomes in critically ill patients with first-ever stroke, revealing significant findings: (i) TyG was significantly related to increased ICU all-cause mortality and hospital all-cause mortality, implying that TyG could be used for mortality risk stratification in the first-ever stroke patients admitted to ICU; (ii) Increased TyG was positively correlated with ICU and hospital length of stay, suggesting ICU/hospital costs and complications might increase; (iii) Elevated TyG was negatively associated with GCS score, eye opening, verbal response, and motor response, indicating that increased TyG was significantly linked to poorer cognitive functioning; and (iv) TyG had promising potential to be applied to ML for predicting ICU all-cause mortality in patients with first-ever stroke, and TyG was important in the ML model.

To our knowledge, no prior study has explored prediction ML models for ICU all-cause mortality in first-ever stroke patients. Our model compares we with other tools for predicting mortality in stroke patients. For example, Chen et al. constructed ML models for predicting 30-day ICU all-cause mortality in stroke patients using 10 clinical features on the first day of ICU admission, with the best performance of LightGBM, which had an AUC of 0.88 in the internal validation cohort [22]. Ouyang et al. constructed ML models for predicting hospital mortality in ICU patients with IS using 18 clinical features [23]. Their best model was RF, with an AUC and F1-score of 0.799 and 0.417 in their internal validation, and with an AUC and F1-score of 0.733 and 0.498 in their external validation, respectively. All of their best models had AUCs lower than 0.9 in the internal validation, and they all also included MV, whereas our model obtained better performance using MV and the other six features. This may be attributed to the fact that TyG is significant for predicting the risk of mortality in stroke patients, nd our model also externally performed well.

Since the calculation of the TyG involves fasting triglycerides and fasting blood glucose, which are closely related to IR. Triglycerides serve as the predominant storage form of fat in the body, with their regulation being influenced by insulin. Increased triglyceride levels are positively correlated with IR [24]. Fasting blood glucose is an important clinical indicator for assessing glucose metabolism status, and it is associated with IR [25]. Guerrero-Romero and colleagues indicated a substantial correlation between the TyG and the total glucose metabolic rate measured by the euglycemic-hyperinsulinemic clamp test (*r* = − 0.681, *P-value* < 0.005), and that the sensitivity and specificity of TyG for the diagnosis of IR were 96.5% and 85.0%, respectively [26]. Moreover, Wu et al. found a moderate correlation (Spearman ρ = 0.51) between the TyG and the homeostatic model assessment of IR, which can also be used to assess IR [27]. Overall, based on prior evidence, TyG could serve as a feasible and easy-to-assess surrogate for IR.

Insulin activates the triglyceride breakdown rate-limiting enzyme in adipose tissue, which promotes the metabolism of triglycerides in chylomicrons and very low-density lipoproteins, thereby helping to lower blood lipids. Evidence suggests that free fatty acids and various adipokines produced by adipose tissue are connected to abnormal insulin signaling, thus adipose tissue has a significant impact on the development of IR [28]. The expansion of adipose tissue triggers the infiltration of macrophages and disrupts the equilibrium between both pro- and anti-inflammatory factors secreted by adipose. This leads to heightened inflammation, compromised insulin sensitivity, and dysregulated lipid metabolism result [29]. The regulation of insulin on blood sugar mainly includes two aspects: promoting glucose uptake in bone iliac muscle, myocardium and adipose tissue, and inhibiting liver glycogenolysis and gluconeogenesis. When these effects are weakened, that is, insulin cannot effectively promote the uptake of glucose by surrounding tissues and cannot inhibit glucose output from the liver, the blood glucose will rise. IR is one of the important factors that promote the occurrence and development of metabolic syndrome, including other risk factors like hyperlipidemia, hypertension, and hyperglycemia. The risk of cardiovascular and cerebrovascular diseases in individuals with metabolic syndrome was 3 times greater than those without metabolic syndrome, and the risk of death was 5–6 times greater than those without metabolic syndrome. Increases the incidence, prevalence and all-cause mortality of type-2 DM placing an overwhelming burden for patients, families and the society, and increasing the total healthcare cost of related diseases by 60% [30]. It has been confirmed that most patients with acute cerebrovascular disease are complicated with hypertension, hyperglycemia, dyslipidemia and many other high-risk factors. These risk factors interact to promote the progression of metabolic disorders in the body. IR not only causes hyperglycemia, hyperlipidemia, hypertension, hyperinsulinemia and other metabolic disorders indirectly leading to large and small vascular diseases, but also directly affects vascular endothelial cells and macrophages and other inflammatory cells, causing endothelial cell dysfunction, blood-brain barrier permeability change, microvasomotor dysfunction, etc. These would further accelerate the progression of atherosclerosis and promote the rupture of atherosclerotic plaque, leading to the incidence of stroke [31]. 

TyG is a potential biomarker indicator for high-risk of all-cause mortality in metabolic disorders, cardiovascular diseases, critically ill patients, infectious diseases [32–34]. However, the precise understanding of the relationship between IR and the occurrence and prognosis of stroke remains limited. From the perspective of pathophysiological mechanisms, the mechanisms behind IR and stroke may be as follows: (i) In a state of impaired IR, there is a decrease in the release of nitric oxide from the vascular endothelium and vasodilatory function, which is linked increased blood pressure and consequential endothelial damage, damage to the vessel wall, and blood vessel fragility, potentially increasing the risk of haemorrhagic stroke; [35] and (ii) IR per se induces myocardial oxidative stress, which inhibits the cardiac antioxidant defence system, leading to platelet aggregation and atherosclerotic thrombosis, and to more cerebrovascular lesions and IS [36]. While monitoring other cardivascular events post-stroke could offer additional insights, the acute nature of ICU admissions limits its utility as a primary outcome. Future studies incorporating post-discharge follow-up could better assess the long-term cardiovascular effects of IR in post-stroke population.

Several previous studies have investigated TyG and stroke occurrence or prognosis, and some have been in the ICU setting. Huo et al. reported TyG and independent risk factors for incident stroke in a middle-aged and older Chinese population (Q1 as reference; Q2: HR 1.40, 95% CI 1.07–1.83; Q3: HR 1.75, 95% CI 1.35–2.27; Q4: HR 1.65, 95% CI 1.27–2.15; adjusted for confounders) [37]. Hu and colleagues showed that in older hypertensive patients, elevated levels of TyG were significantly associated to the first-ever stroke (Q1 as the reference group, Q4: HR 1.90, 95% CI 1.04–3.45) or first-ever IS (Q1 as the reference group, Q4: HR 2.45, 95% CI 1.16–5.20) [38]. However, their 95% CIs were wide, given the limited sample size and stroke/IS events, thus the association of TyG with first-ever stroke occurrence needs to be further explored. For all-cause mortality post-stroke, one recent study using the MIMIC-IV database showed that higher TyG was strongly correlated with ICU all-cause mortality (HR 1.65, 95% CI 1.24–2.20) and hospital all-cause mortality (HR 1.37, 95% CI 1.05–1.78) in severely ill IS patients [39], and TyG was linked to elevated risks of 30-day all-cause mortality (HR 1.32, 95% CI 1.05–1.67), 90-day all-cause mortality (HR 1.27, 95% CI 1.04–1.55) and 1-year all-cause mortality (HR 1.22, 95% CI 1.03–1.44) [40]. 

For other prognostic outcomes post-stroke, Lee et al. demonstrated that high levels of TyG were significantly related to poor three-month functional result (modified Rankin Scale ≥ 3) in acute IS patients receiving reperfusion therapy (OR 5.22, 95% CI 1.39–19.57), early neurological deterioration occurred in approximately 20% of the high TyG group, whereas this did not occur in the low TyG group [41]. We could not collect post-stroke functional scores in our study, but we indicated that TyG was significantly correlated with GCS scores, similarly suggesting that TyG was associated with early neurological deterioration. Moreover, Cheng et al. suggested that TyG was negatively correlated with cognitive impairment scores, Montreal Cognitive Assessment scores, in post-acute IS patients (*r* = − 0.272, *P* < 0.001), elevated levels of TyG correlated with occurrence of cognitive impairment 3-month post-stroke [42]. However, no prior study exists on TyG and prognosis of patients with first-ever critical ill stroke.

Therefore, our study adds to this research gap by demonstrating the important role of IR based on TyG assessment in the prognostic management (especially all-cause mortality and neurological abnormalities) of first-ever stroke in the ICU, to optimise treatment options and improve treatment efficiency.

### Strengths and limitations

This study provided several important strengths. First, the study population was critically ill patients with first-ever stroke, which is different from the scope of previous studies that focused on all critically ill stroke patients, making our results more relevant and clinically applicable. Second, this study not only assessed hospital and ICU all-cause mortality, but also firstly included conscious status as a prognostic outcome, comprehensively analysing the impact of TyG on the clinical prognosis of ICU patients with first-ever stroke. Third, this study also constructed the first ML prediction model for ICU all-cause mortality in first-ever stroke patients based on TyG and other features, and it also performed well in an independent external cohort. Therefore, these findings further extend the use of TyG in the prognostic assessments of critically ill patients with first-ever stroke and provide a new basis for early clinical identification of high-risk patients.

Notably, there were several unavoidable limitations to our investigation. First, since the study was retrospective in nature, selection bias may exist and we may have missed some potential confounders (e.g. CRP) for our outcomes of interest. Second, there were substantial numbers of unspecified stroke diagnosis records in this study, which resulted in our inability to accurately assess the association of TyG for different stroke types with our outcome of interest. Third, we identified ‘first-ever stroke’ cases by excluding patients with recorded prior strokes. Due to limitations of EICU, we could not exclude patients with silent strokes, which are asymptomatic and often detected only through imaging. Silent strokes may affect baseline risk and outcomes, introducing potential bias. Future prospective studies should differentiate between symptomatic and silent strokes for more accurate risk assessment in first-ever stroke patients. Fourth, the TyG calculated in our study was derived from the initial triglyceride and blood glucose levels on ICU admission, without adherence to strict fasting conditions. However, in the external cohort, we collected blood glucose and triglycerides under strict fasting conditions and still showed a similar association of TyG with ICU all-cause mortality and conscious status, but it is still necessary to further assess whether the association between TyG calculated under fasting conditions and outcome would be significantly different in a larger cohort. Fifth, the EICU database lacked detailed pre-hospital medication records, preventing us from excluding patients on prior treatments for dyslipidemia or diabetes. While we included medication data during hospitalisation (statins, fibrates, insulin, and oral hypoglycemic agents), the absence of historical data may affect the associations observed, and future prospective studies should include complete medication histories. Sixth, stroke severity significantly impacts patient prognosis, however, the EICU database lacks such specific data. Future studies are needed to access the comprehensive data of stroke severity. Seventh, we did not consider whether changes in TyG would have other effects on outcomes, and thus further evaluation of the association of dynamically changing TyG with the prognosis of critically ill patients with first-ever stroke is necessary.

## Conclusions

TyG was identified as an independent risk factor for ICU and hospital all-cause mortality in patients with first-ever stroke, with an optimal cut-off of 9.265. This threshold may help clinicians identify patients at higher risk earlier, allowing for more aggressive management strategies. Moreover, the ML model incorporating TyG demonstrated excellent predictive performance in external validation, highlighting the significance of IR (measured by TyG) in the prognostic assessment and early risk stratification of critically ill patients with first-ever stroke.

## Electronic supplementary material

Below is the link to the electronic supplementary material.

Supplementary Material 1

## Data Availability

No datasets were generated or analysed during the current study.

## Abbreviations

IR: Insulin resistance
TyG: Triglyceride-glucose index
EICU: eICU Collaborative Research Database
APACHE IV: Acute Physiology and Chronic Health Evaluation IV
MV: Mechanical ventilation
RCS: Restricted cubic spline
RF: Random forest
DCA: Decision curve analysis
SHAP: SHapley Additive exPlanation
