# Supplementary material for: Exploring the prognostic impact of triglyceride-glucose index in critically ill patients with first-ever stroke: insights from traditional methods and machine learning-based mortality prediction
Source: Cardiovasc Diabetol. 2024 Dec 18;23:443. doi: 10.1186/s12933-024-02538-y (PMC11658255; doi:10.1186/s12933-024-02538-y)
Supplement: Supplementary file 1 — Supplementary Material 1 [file 12933_2024_2538_MOESM1_ESM.pdf]

Supplementary Table S1. Proportion of missing values for the variable in the EICU.

**Variables**	**n (%)**
Height	52 (1.64%)
Weight	53 (1.67%)
LDL-C	801 (25.24%)
HDL-C	70 (2.21%)
CRP	3081 (97.10%)
RDW	561 (17.68)
APACHE IV	286 (9.01%)

Abbreviations: APACHE IV, Acute Physiology and Chronic Health Evaluation IV; CRP, C-reactive protein; EICU, eICU Collaborative Research Database; HDL-C, high-density lipoprotein cholesterol; LDL-C, low-density lipoprotein cholesterol; RDW, red cell distribution width.

Supplementary Table S2. Baseline characteristics of participants categorized by quartiles of TyG in the EICU.

	TyG < 9.265	TyG ≥ 9.265	*P-value*
N	2436	737	
Age, years	69.00 (57.00, 80.00)	64.00 (54.00, 73.00)	< 0.001
Male, n (%)	1254 (51.5%)	410 (55.6%)	0.048
Body mass index, kg/m^2	27.11 (23.74, 31.66)	29.88 (26.33, 34.55)	< 0.001
Race, n (%)			0.073
African, American	285 (11.7%)	83 (11.3%)	
Caucasian	1841 (75.6%)	536 (72.7%)	
Other/Unknow	310 (12.7%)	118 (16.0%)	
APACHE IV	46.00 (34.00, 60.00)	48.00 (36.00, 69.00)	< 0.001
Comorbidities, n (%)			
Diabetes	171 (7.0%)	141 (19.1%)	< 0.001
Hypertension	747 (30.7%)	255 (34.6%)	0.047
Coronary artery disease	65 (2.7%)	25 (3.4%)	0.311
Chronic kidney disease	72 (3.0%)	30 (4.1%)	0.152
Heart failure	73 (3.0%)	15 (2.0%)	0.200
Myocardial infarction	39 (1.6%)	25 (3.4%)	0.004
Atrial fibrillation	258 (10.6%)	56 (7.6%)	0.017
Respiratory failure	329 (13.5%)	165 (22.4%)	< 0.001
Acute kidney injury	92 (3.8%)	47 (6.4%)	0.004
Cirrhosis	8 (0.3%)	2 (0.3%)	0.809
Sepsis	43 (1.8%)	39 (5.3%)	< 0.001
Laboratory results at first day			
LDL-C, mg/dL	86.00 (66.00, 111.00)	99.00 (71.00, 127.00)	< 0.001
HDL-C, mg/dL	46.00 (37.00, 57.00)	36.00 (29.00, 44.00)	< 0.001
RDW, %	13.80 (13.20, 14.80)	13.80 (13.10, 14.65)	0.096
Triglycerides, mg/dL	90.00 (67.75, 118.00)	197.00 (150.00, 266.00)	< 0.001
Glucose, mg/dL	114.00 (98.00, 135.00)	168.00 (129.00, 239.00)	< 0.001
Medications, n (%)			
Antiplatelet agents	244 (10.0%)	93 (12.6%)	0.048
Anticoagulants	158 (6.5%)	39 (5.3%)	0.258
Vasopressors	103 (4.2%)	44 (6.0%)	0.002
Statins	142 (5.8%)	56 (7.6%)	0.083
Fibrates	2 (0.1%)	0 (0.0%)	1.000
Insulin	298 (12.2%)	193 (26.2%)	< 0.001
Oral hypoglycaemic agents	1 (0.1%)	1 (0.1%)	0.411
Interventions, n (%)			
Thrombolysis	574 (23.6%)	173 (23.5%)	0.960
Mechanical ventilation	367 (15.1%)	215 (29.2%)	< 0.001

Abbreviations: APACHE IV, Acute Physiology and Chronic Health Evaluation IV; EICU, eICU Collaborative Research Database; HDL-C, high-density lipoprotein cholesterol; LDL-C, low-density lipoprotein cholesterol; RDW, Red cell distribution width.

Supplementary Table S3. Outcomes of participants categorized by TyG in the EICU.

	ALL	TyG < 9.265	TyG ≥ 9.265	*P-value*
ICU mortality, n (%)	181 (5.7%)	99 (4.1%)	82 (11.1%)	< 0.001
Hospital mortality, n (%)	354 (11.2%)	223 (9.2%)	131 (17.8%)	< 0.001
ICU length of stay, days	1.93 (1.17, 3.80)	1.91 (1.13, 3.58)	2.03 (1.30, 4.67)	< 0.001
Hospital length of stay, days	4.86 (2.84, 9.00)	4.83 (2.83, 8.96)	4.95 (2.86, 9.70)	0.301
GCS score*	15.00 (12.00, 15.00)	15.00 (12.00, 15.00)	14.00 (10.00, 15.00)	< 0.001
Eye opening*	4.00 (4.00, 4.00)	4.00 (4.00, 4.00)	4.00 (3.00, 4.00)	< 0.001
Verbal response*	5.00 (3.00, 5.00)	5.00 (3.00, 5.00)	5.00 (1.00, 5.00)	< 0.001
Motor response*	6.00 (6.00, 6.00)	6.00 (6.00, 6.00)	6.00 (5.00, 6.00)	< 0.001

*Because some patients were not assessed for GCS, this part of the sample was: N = 2957.

Abbreviations: GCS, Glasgow Coma Score; EICU, eICU Collaborative Research Database; ICU, intensive care unit; TyG, triglyceride-glucose index.

Supplementary Table S4. The association between TyG and secondary outcomes in the EICU.

	TyG			
	**r**	***P-value***	**β**	***P-value***
Length of ICU stay	0.077	< 0.001	0.092	< 0.001
Length of hospital stay	0.042	0.021	0.054	0.002
GCS score*	- 0.132	< 0.001	- 0.131	< 0.001
Eye opening*	- 0.128	< 0.001	- 0.128	< 0.001
Verbal response*	- 0.107	< 0.001	- 0.113	< 0.001
Motor response*	- 0.116	< 0.001	- 0.106	< 0.001

*Because some patients were not assessed for GCS, this part of the sample was: N = 2957.

Abbreviations: EICU, eICU Collaborative Research Database; GCS, Glasgow Coma Score; ICU, intensive care unit; TyG, triglyceride-glucose index.

Supplementary Table S5. Sensitivity analyses of associations between TyG and mortality outcomes in the first-ever stroke patients in the EICU after additional adjusting for RDW.

	**HR (95% CI)**	***P-value***
**ICU all-cause mortality**		
Continuous TyG	1.34 (1.07, 1.68)	0.010
Categorised TyG		
TyG < 9.265	*Reference*	
TyG ≥ 9.265	1.94 (1.39, 2.69)	< 0.001
**Hospital all-cause mortality**		
Continuous TyG	1.33 (1.12, 1.57)	< 0.001
Categorised TyG		
TyG < 9.265	*Reference*	
TyG ≥ 9.265	1.75 (1.36, 2.23)	< 0.001

Adjusted by Model 3 and RDW.

Abbreviations: CI, confidence interval; EICU, eICU Collaborative Research Database; HR, hazard ratio; ICU, intensive care unit; RDW, red cell distribution width; TyG, triglyceride-glucose Index.

Supplementary Table S6. Sensitivity analyses of associations between TyG and mortality outcomes in the first-ever stroke patients in the EICU after additional adjusting for lipid lowering therapy.

	**HR (95% CI)**	***P-value***
**ICU mortality**		
Continuous TyG	1.34 (1.07, 1.67)	0.012
Categorised TyG		
TyG < 9.265	*Reference*	
TyG ≥ 9.265	1.93 (1.39, 2.69)	< 0.001
**Hospital mortality**		
Continuous TyG	1.30 (1.10, 1.54)	0.001
Categorised TyG		
TyG < 9.265	*Reference*	
TyG ≥ 9.265	1.70 (1.33, 2.17)	< 0.001

Adjusted by Model 3 and lipid lowering therapy (statins and fibrates).

Abbreviations: CI, confidence interval; EICU, eICU Collaborative Research Database; HR, hazard ratio; ICU, intensive care unit; TyG, triglyceride-glucose Index.

Supplementary Table S7. Sensitivity analyses of associations between TyG and mortality outcomes in the first-ever stroke patients in the EICU after additional adjusting for hypoglycaemic therapy.

	HR (95% CI)	*P-value*
**ICU mortality**		
Continuous TyG	1.33 (1.06, 1.66)	0.014
Categorised TyG		
TyG < 9.265	*Reference*	
TyG ≥ 9.265	1.90 (1.37, 2.64)	< 0.001
**Hospital mortality**		
Continuous TyG	1.29 (1.09, 1.53)	0.003
Categorised TyG		
TyG < 9.265	*Reference*	
TyG ≥ 9.265	1.68 (1.31, 2.14)	< 0.001

Adjusted by Model 3 and hypoglycaemic therapy (insulin and oral hypoglycaemic agents).

Abbreviations: CI, confidence interval; EICU, eICU Collaborative Research Database; HR, hazard ratio; ICU, intensive care unit; TyG, triglyceride-glucose Index.

Supplementary Table S8. Sensitivity analyses of associations between adjustment factors and outcomes in the first-ever stroke patients in the EICU.

	ICU all-cause mortality		Hospital all-cause nortality		Glasgow Coma Scale	
**Adjustment factors**	**HR (95% CI)**	***P-value***	**HR (95% CI)**	***P-value***	**β (95% CI)**	***P-value***
Age	1.01 (1.00, 1.02)	0.272	1.02 (1.01, 1.02)	< 0.001	-0.01 (-0.01, 0.02)	0.162
Sex	1.47 (1.09, 1.99)	0.001	1.23 (1.00, 1.52)	0.056	0.17 (-0.07, 0.41)	0.159
Race					0.21 (0.04, 0.37)	0.015
Other/Unknow	*Reference*		*Reference*			
African American	1.55 (0.89, 2.70)	0.121	1.07 (0.71, 1.62)	0.733		
Caucasian	1.36 (0.86, 2.14)	0.185	1.24 (0.91, 1.69)	0.182		
Body mass index	0.99 (0.97, 1.01)	0.241	0.99 (0.98, 1.00)	0.169	0.02 (0.00, 0.03)	0.029
APACHE IV	1.03 (1.03, 1.04)	< 0.001	1.03 (1.03, 1.04)	< 0.001	-0.09 (-0.09, -0.09)	< 0.001
Diabetes mellitus	0.85 (0.54, 1.33)	0.470	1.32 (0.98, 1.77)	0.065	-0.50 (-0.89, -0.11)	0.012
Hypertension	0.72 (0.52, 0.98)	0.036	0.92 (0.74, 1.14)	0.461	-0.31 (-0.56, -0.06)	0.016
Coronary artery disease	1.43 (0.70, 2.90)	0.325	1.56 (0.96, 2.54)	0.074	0.16 (-0.53, 0.85)	0.648
Chronic kidney disease	0.79 (0.35, 1.79)	0.580	1.38 (0.87, 2.19)	0.176	-0.52 (-1.18, 0.13)	0.119
Heart failure	0.97 (0.46, 2.07)	0.937	1.00 (0.59, 1.71)	1.000	-1.15 (-1.86, -0.44)	0.002
Myocardial infarction	1.00 (0.47, 2.13)	0.999	0.99 (0.54, 1.82)	0.981	-0.37 (-1.22, 0.48)	0.397
Atrial fibrillation	0.71 (0.42, 1.21)	0.212	0.96 (0.68, 1.33)	0.786	-0.48 (-0.87, -0.09)	0.017
Respiratory failure	1.91 (1.40, 2.59)	< 0.001	2.54 (2.04, 3.16)	< 0.001	-4.38 (-4.67, -4.10)	< 0.001
Acute kidney injury	1.04 (0.63, 1.73)	0.869	1.50 (1.05, 2.13)	0.027	-1.78 (-2.35, -1.21)	< 0.001
Cirrhosis	3.06 (1.13, 8.26)	0.028	5.57 (2.76, 11.20)	< 0.001	-3.39 (-5.42, -1.36)	0.001
Sepsis	0.84 (0.45, 1.55)	0.569	1.39 (0.91, 2.13)	0.128	-2.63 (-3.40, -1.87)	< 0.001
HDL-C	0.99 (0.98, 1.00)	0.007	0.99 (0.99, 1.00)	0.016	0.00 (-0.01, 0.01)	0.361
LDL-C	1.00 (0.99, 1.00)	0.012	0.99 (0.99, 1.00)	< 0.001	0.01 (0.01, 0.01)	< 0.001

Red cell distribution width	1.06 (0.99, 1.13)	0.082	1.10 (1.06, 1.15)	< 0.001	-0.21 (-0.27, -0.14)	< 0.001
Antiplatelet agents	0.60 (0.35, 1.04)	0.067	0.66 (0.45, 0.97)	0.033	-0.01 (-0.39, 0.37)	0.961
Anticoagulants	0.59 (0.30, 1.15)	0.122	0.53 (0.31, 0.88)	0.015	0.28 (-0.22, 0.77)	0.269
Vasopressors	1.45 (0.96, 2.18)	0.076	1.88 (1.37, 2.57)	< 0.001	-2.26 (-2.81, -1.71)	< 0.001
Statins	0.63 (0.31, 1.29)	0.208	0.58 (0.34, 0.99)	0.045	0.35 (-0.14, 0.84)	0.166
Fibrates	0.05 (0.00, $8.75e^{23}$)	0.919	0.05 (0.00, $6.63e^{10}$)	0.833	2.12 (-2.41, 6.66)	0.359
Insulin	1.15 (0.81, 1.65)	0.433	1.28 (0.99, 1.65)	0.060	-0.48 (-0.81, -0.15)	0.004
Oral hypoglycaemic agents	0.05 (0.00, $1.51e^{16}$)	0.884	0.50 (0.00, $2.08e^{9}$)	0.810	2.12 (-2.41, 6.66)	0.359
Thrombolysis	0.61 (0.36, 1.02)	0.062	0.65 (0.47, 0.89)	0.008	1.18 (0.90, 1.45)	< 0.001
Mechanical ventilation	4.48 (3.22, 6.23)	< 0.001	4.23 (3.41, 5.24)	< 0.001	-5.22 (-5.46, -4.97)	< 0.001

Abbreviations: APACHE IV, Acute Physiology and Chronic Health Evaluation IV; CI, confidence interval; EICU, eICU Collaborative Research Database; HDL-C, high-density lipoprotein cholesterol; HR, hazard ratio; ICU, intensive care unit; LDL-C, low-density lipoprotein cholesterol.

Supplementary Table S9. Best hyperparameters of each classifier.

Classifiers	Hyperparameters	
LightGBM	n_estimators	151
	max_depth	5
	learning_rate	0.01
	boosting_type	gbdt
	objective	binary
	num_leaves	31
	colsample_bytree	0.7707904788350928
	min_child_samples	132
	subsample	0.8699333119864081
	class_weight	'balanced'
Random Forest	n_estimators	79
	max_depth	6
	criterion	'entropy'
	min_samples_leaf	30
	random_state	64
	class_weight	'balanced'
	min_samples_split	2
Logistic Regression	C	10
	penalty	l2
	solver	'saga'
	max_iter	100
	class_weight	'balanced'
Support Vector Machine	C	1
	kernel	'rbf'
	probability	True
	tol	0.0001
	gamma	‘auto’
	class_weight	'balanced'
Multilayer Perceptron	solver	'adam'
	activation	'relu'
	hidden_layer_sizes	[5,]
	alpha	0.001
	max_iter	300
	random_state	3
Gaussian Naive Bayes	var_smoothing	1e-09
K-nearest Neighbors	n_neighbors	24

	p	1
	metric	minkowski
	weights	Uniform

Abbreviations: LightGBM, light gradient boosting machine.

Supplementary Table S10. Baseline characteristics of patients in the *external validation cohort*.

	ALL (N = 201)	ICU survivors (N = 175)	ICU non-survivors (N = 26)	*P-value*
Age, years	71.00 (63.00, 77.00)	71.00 (62.00, 77.00)	70.50 (67.00, 76.00)	0.967
Male, n (%)	130 (64.7)	109 (62.3)	21 (80.8)	0.080
TyG	9.00 (8.59, 9.38)	8.96 (8.54, 9.32)	9.30 (9.00, 9.69)	0.002
HDL-C, mg/dL	36.74 (27.07, 45.05)	36.74 (26.68, 45.63)	35.96 (29.39, 42.34)	0.757
LDL-C, mg/dL	95.13 (65.16, 122.20)	95.13 (63.03, 120.97)	94.16 (76.76, 120.55)	0.464
Respiratory failure, n (%)	85 (42.3)	68 (38.9)	17 (65.4)	0.018
Mechanical ventilation, n (%)	77 (38.3)	61 (34.9)	16 (61.5)	0.016
Vasopressor, n (%)	46 (22.9)	22 (12.6)	24 (92.3)	< 0.001

Abbreviations: HDL-C, high-density lipoprotein cholesterol; LDL-C, low-density lipoprotein cholesterol.

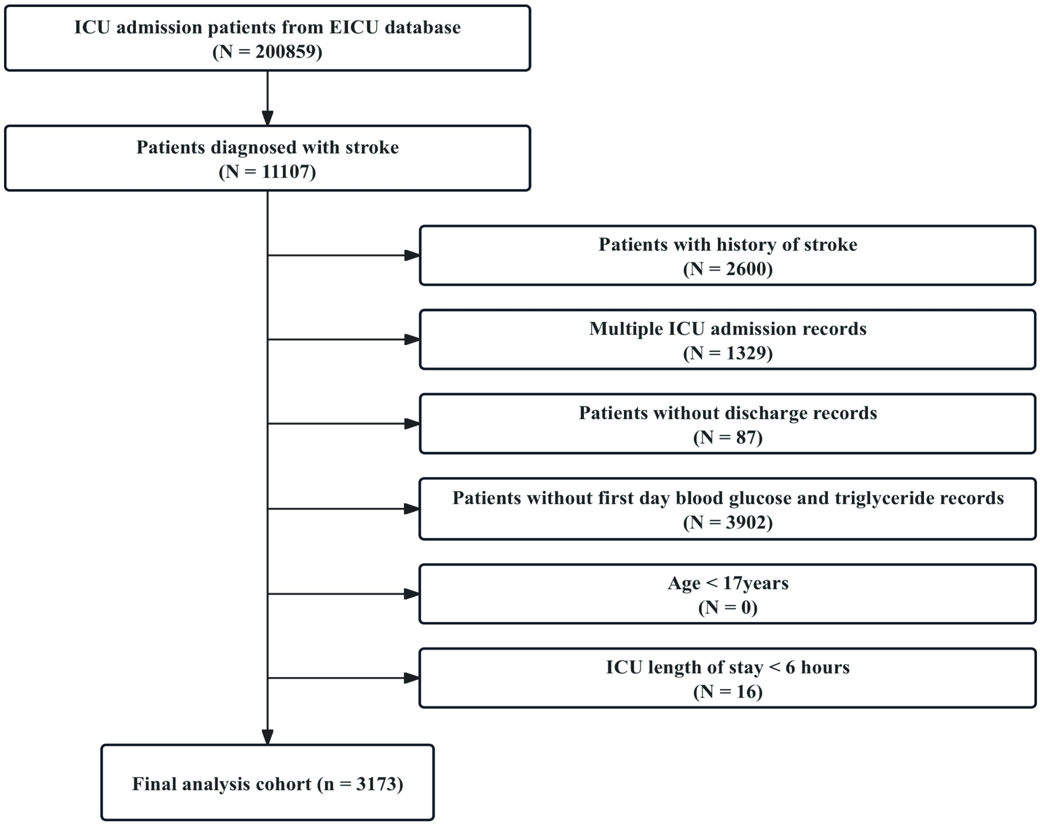

Supplementary Fig. S1 Flowchart of this study. EICU, eICU Collaborative Research Database; ICU, intensive care unit.

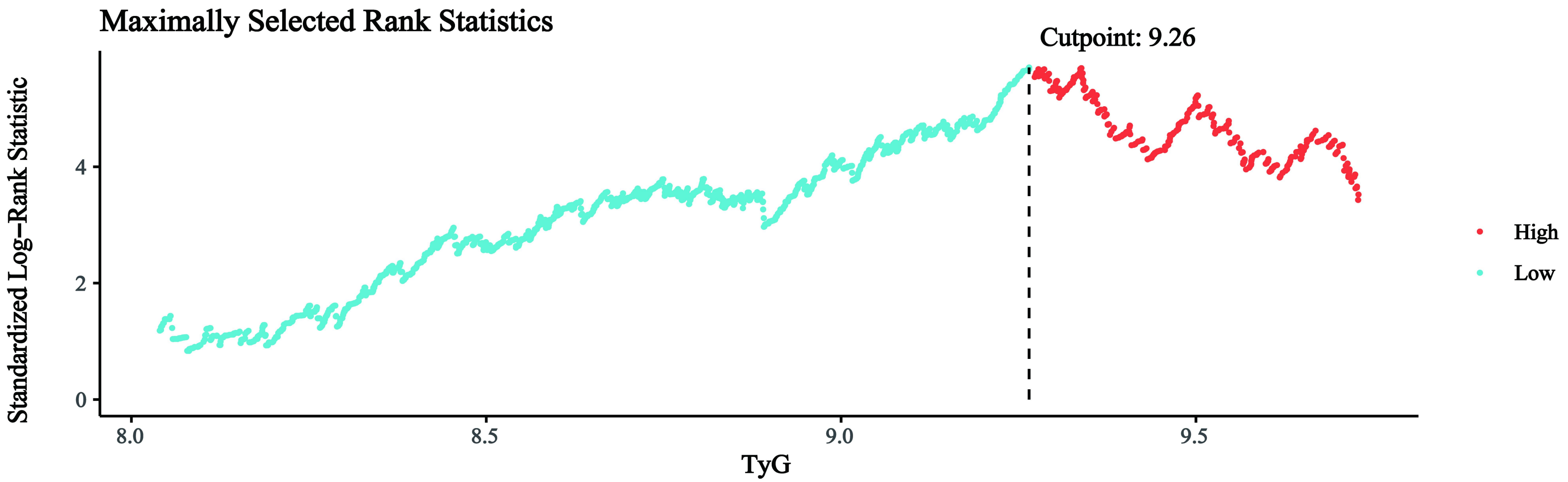

Supplementary Fig. 2 Visualisation of the optimal cut-off point for TyG for ICU all-cause mortality in the EICU. EICU, eICU Collaborative Research Database; ICU, intensive care unit; TyG, triglyceride-glucose index.

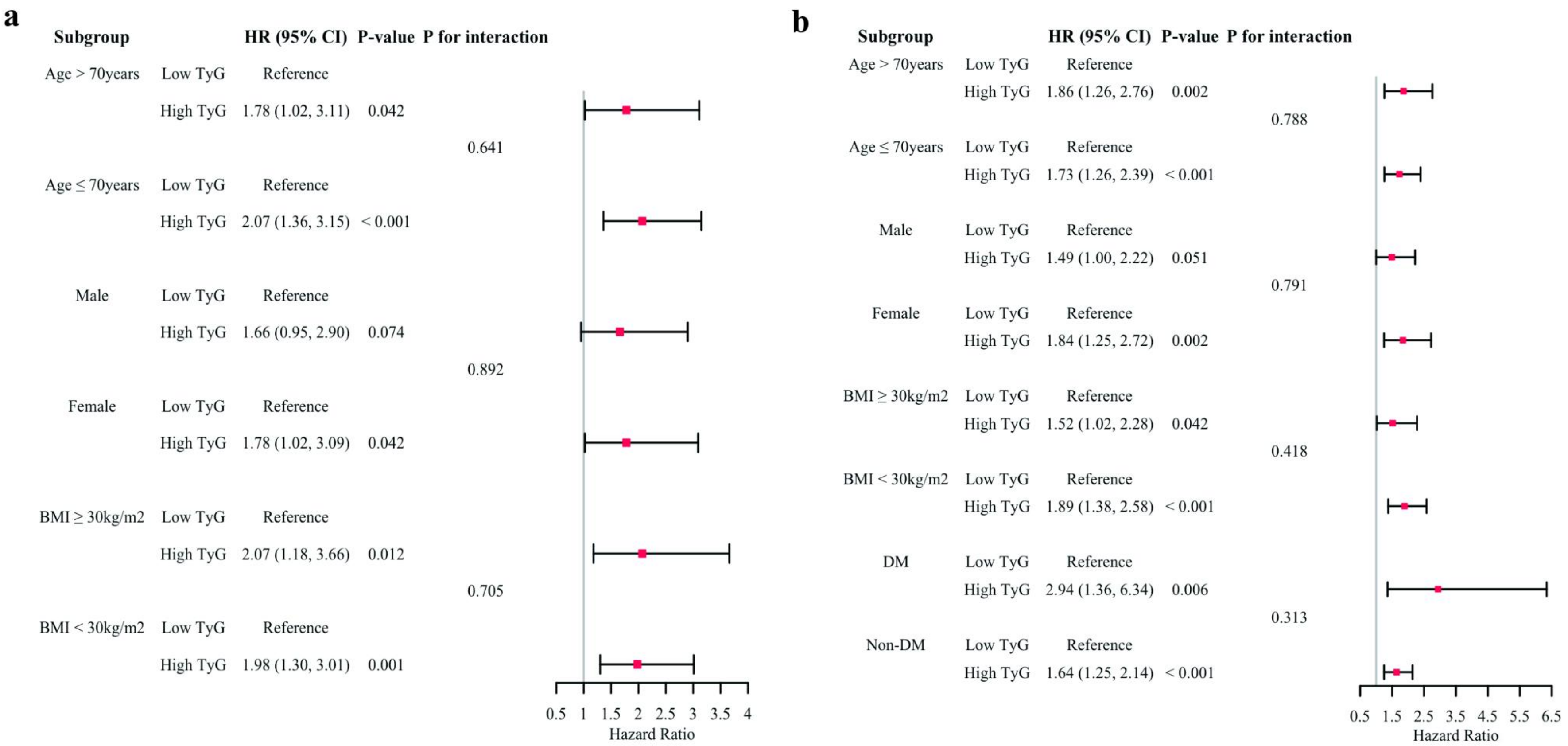

Supplementary Fig. S3 Subgroup analysis of continious TyG and ICU mortality (a) and hospital mortality (b) in the EICU. BMI, body mass index; CI, confidence interval; EICU, eICU Collaborative Research Database; ICU, intensive care unit; OR, odds ratio; TyG, triglyceride-glucose index.

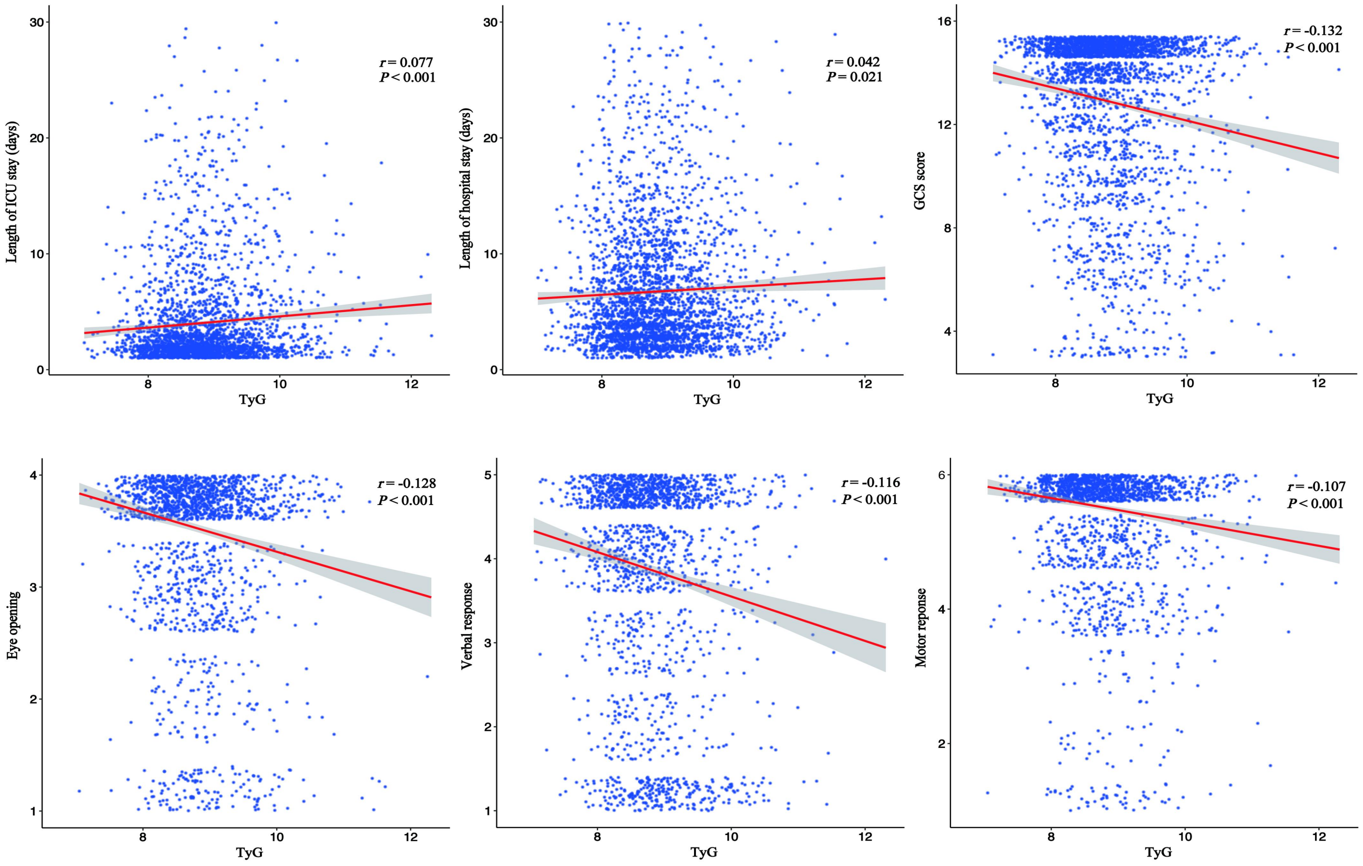

Supplementary Fig. S4 Scatter plots of TyG with length of ICU stay, length of hospital stay, GCS and its compositions in the EICU.
EICU, eICU Collaborative Research Database; GCS, Glasgow Coma Score; ICU, intensive care unit; TyG, triglyceride-glucose index.

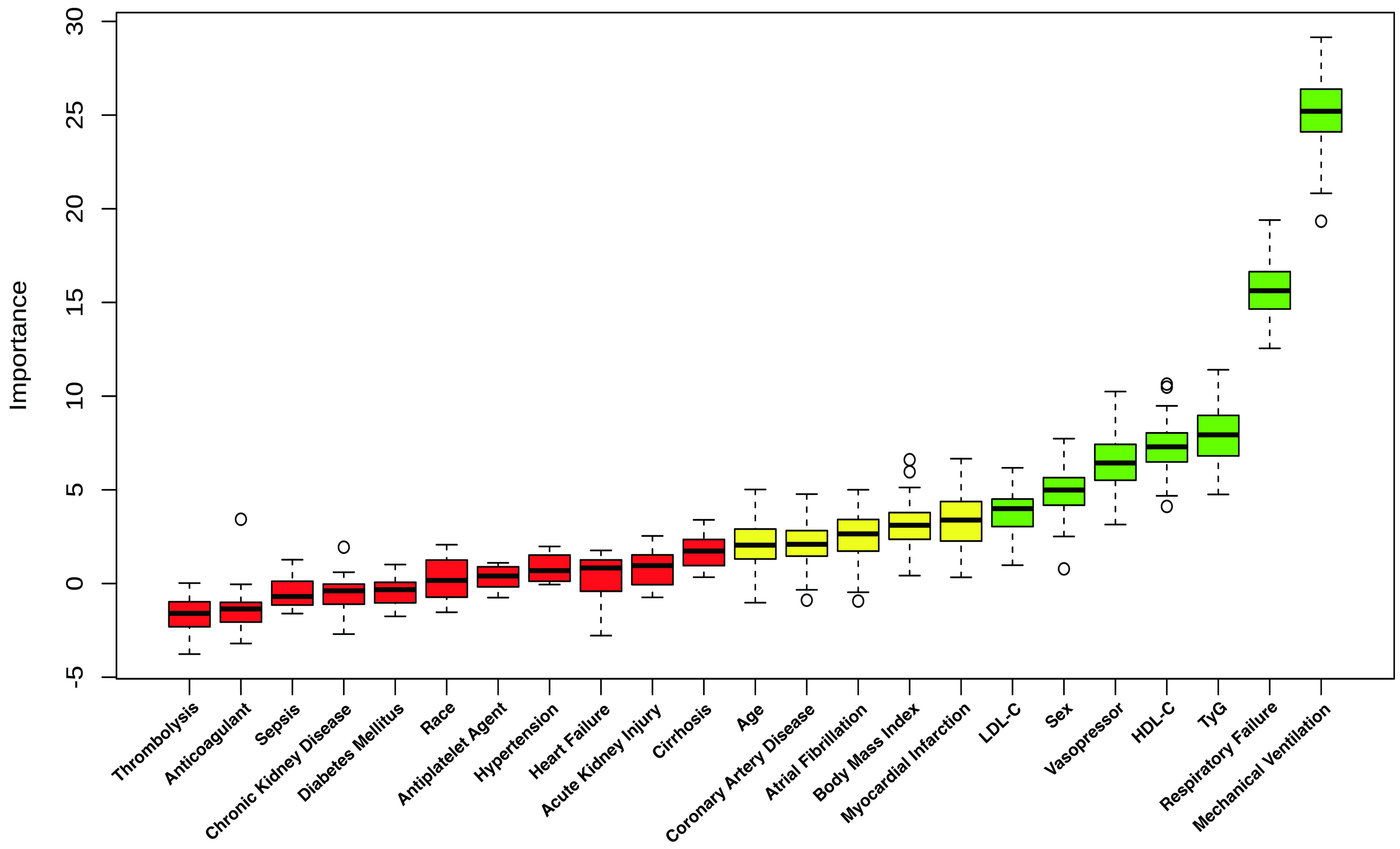

Supplementary Fig. S5 Boruta feature importance ranking for predicting ICU all-cause mortality in patients diagnosed with first-ever stroke. HDL-C, high-density lipoprotein cholesterol; LDL-C, low-density lipoprotein cholesterol; ICU, intensive care unit; TyG, triglyceride-glucose index.

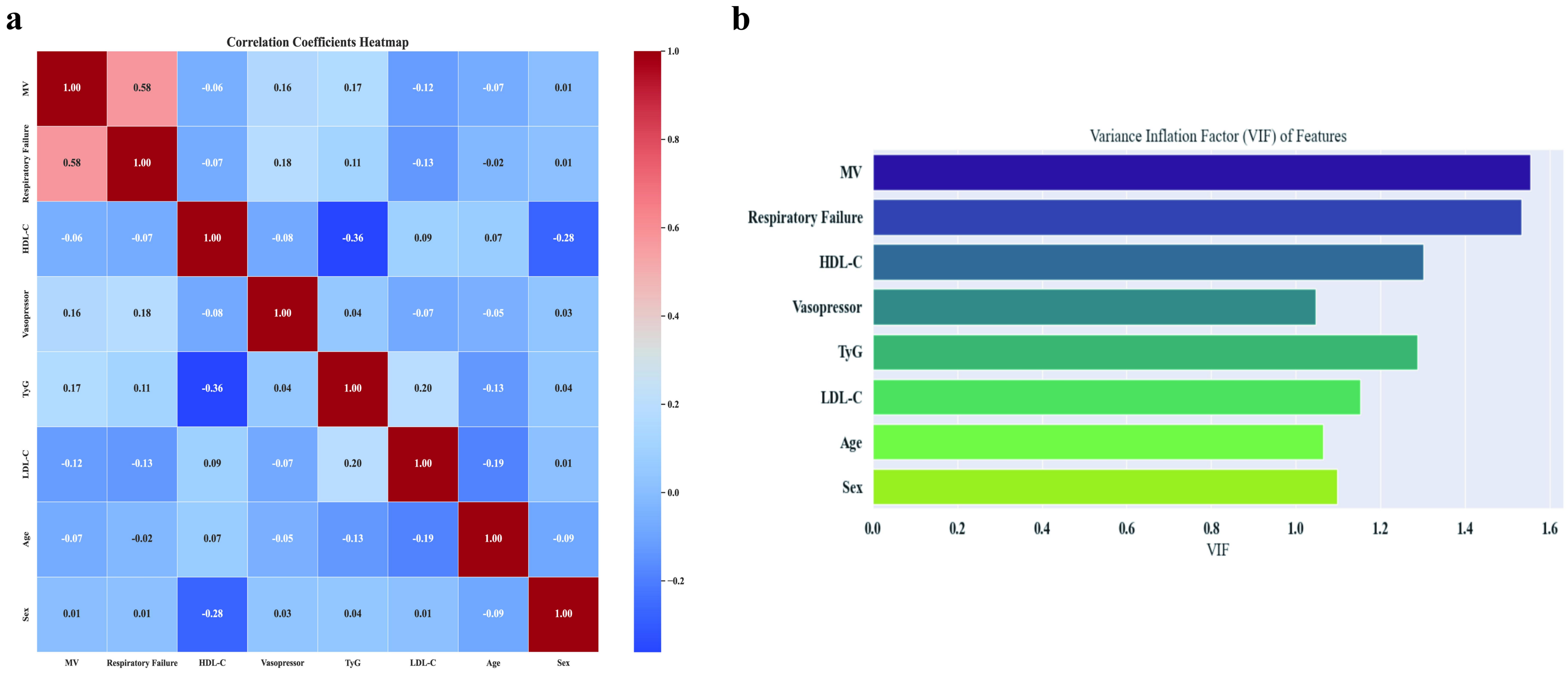

Supplementary Fig. S6. Pearson's correlation test and variance inflation factor test among selected features.

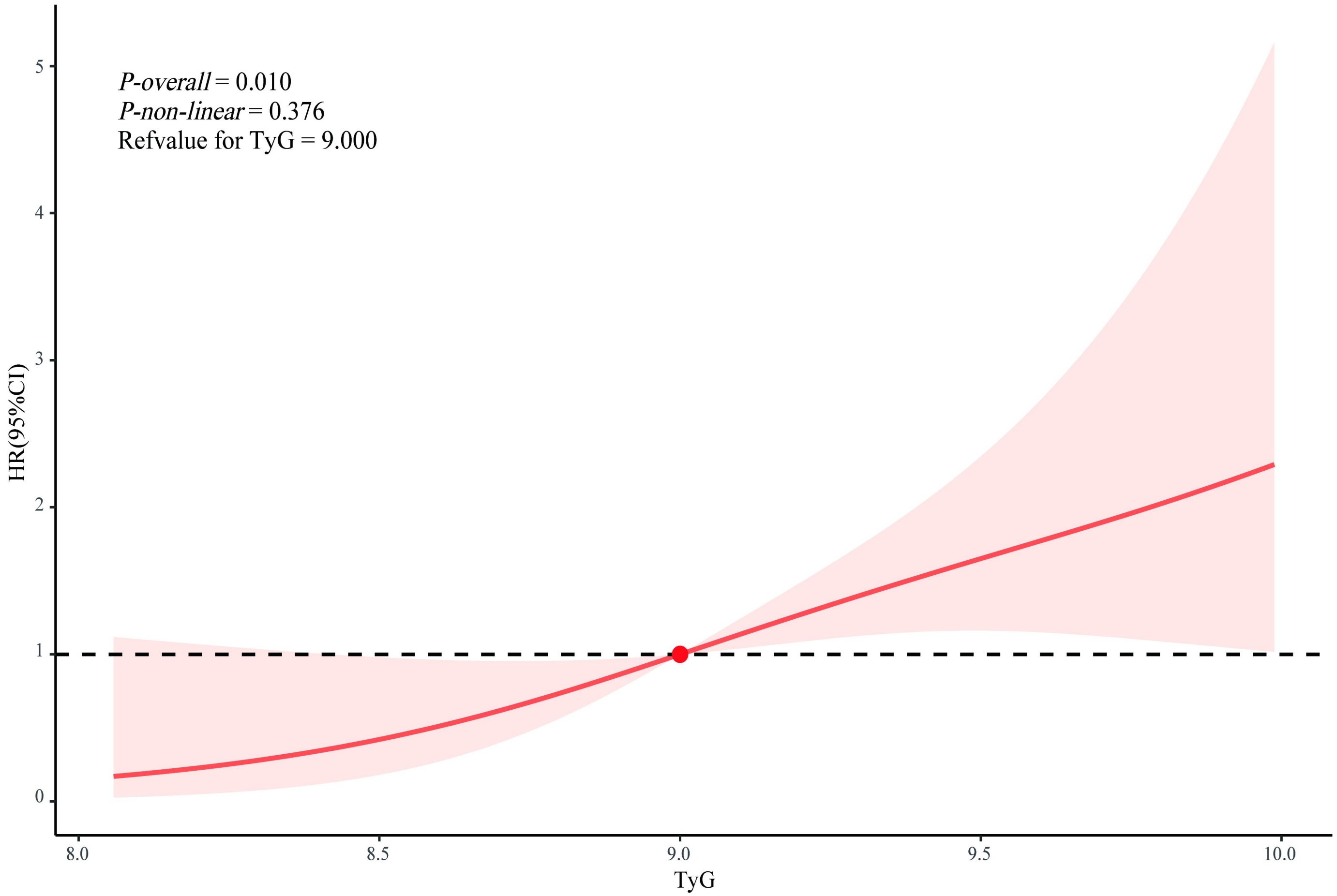

Supplementary Fig. S7 Association between continious TyG and ICU mortality in the external validation cohort. CI, confidence interval; ICU, intensive care unit; OR, odds ratio; TyG, triglyceride-glucose index.

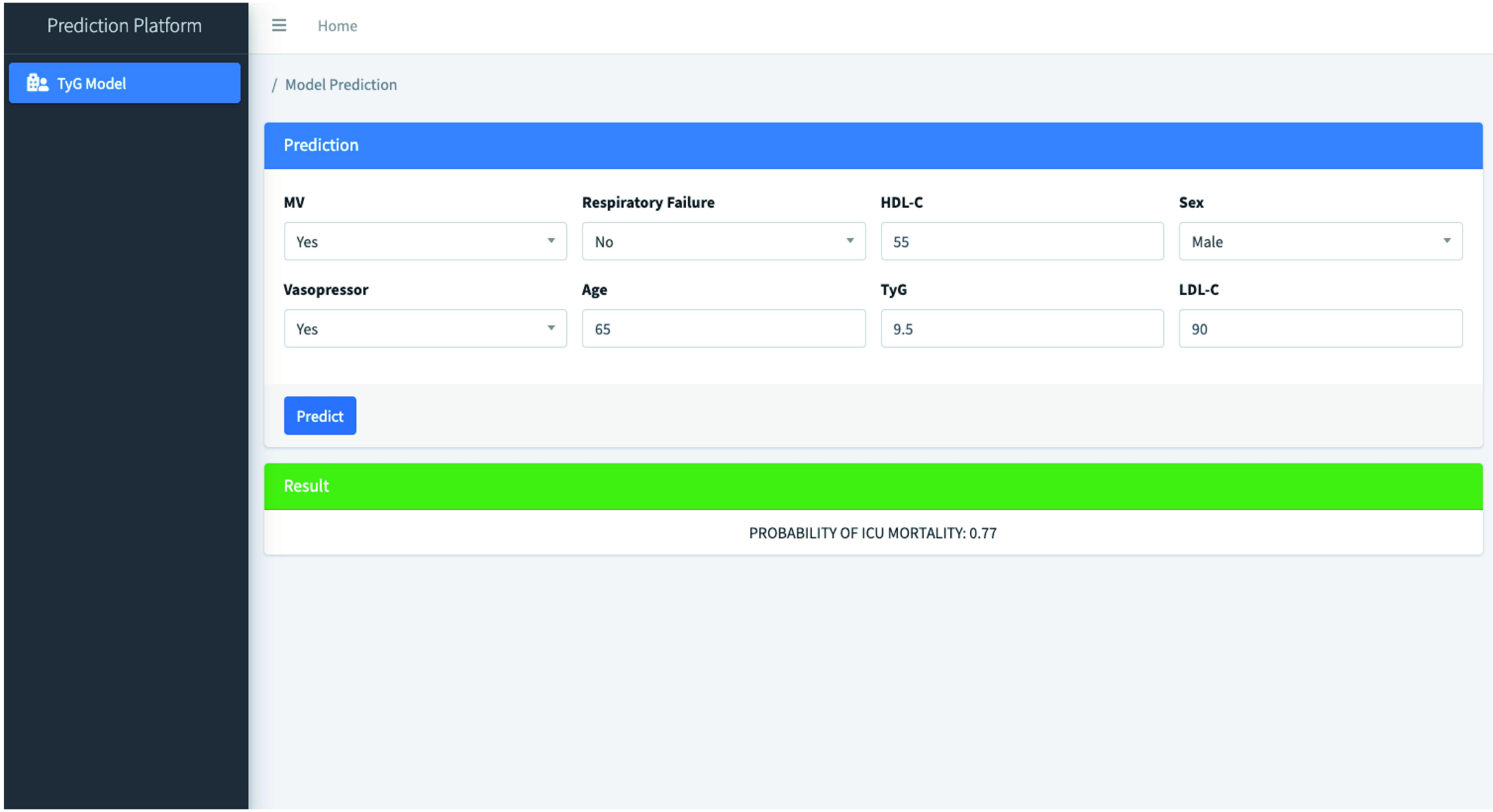

Supplementary Fig. S8 The interface of the web platform embedded with the random forest model. The units of LDL-C and HDL-C are mg/dL. HDL-C, high-density lipoprotein cholesterol; LDL-C, low-density lipoprotein cholesterol; ICU, intensive care unit; MV, mechanical ventilation; TyG, triglyceride-glucose index.
